# Effect of a trauma proforma on the quality of documentation in the Emergency Department

**DOI:** 10.1186/cc9874

**Published:** 2011-03-11

**Authors:** JE Millar, B Fisher, R McLaughlin

**Affiliations:** 1 Royal Victoria Hospital, Belfast, UK

## Introduction

Improvements in trauma care are driven by an understanding of the patient population and their outcome after injury. In order to achieve this, many centres submit data to trauma registries. This process relies on accurate and comprehensive documentation. The aim of this study was to evaluate whether the introduction of a standardised proforma for major trauma patients improved the quality of documentation in an urban Emergency Department (ED).

## Methods

In September 2010 a proforma was introduced within the ED for use in patients presenting after major trauma. Prior to this, clinical documentation for these patients was recorded on the standard ED record. The last 30 patients attending the ED prior to introduction of the proforma, with major injuries requiring admission to a critical care bed or the operating room, were identified. In addition, 15 completed proformas were available for comparison. In order to set a standard for this comparison, the Utstein template for uniform reporting of data following major trauma [1] was examined to identify core variables that should reasonably be recorded in the ED. Of 31 variables, 23 were felt to be relevant. The ED record or proforma for each patient was then scrutinised in an attempt to extract these variables. A comparison was made between the two groups.

## Results

In those attending prior to the introduction of a proforma, the mean number of variables identifiable from the clinical record was 12/23 (52%); after the introduction of the proforma this improved to 20/23 (87%). Several parameters were well documented amongst both groups including age, gender and those relating to mechanism of injury (accountable for 5/23 variables). These were identifiable in greater than 95% of cases. Use of the proforma improved the documentation of the remaining 18 variables, including base excess, level of prehospital care and first key emergency intervention. The most marked improvements were seen in the documentation of prehospital observations (GCS, GCS motor component, systolic blood pressure and respiratory rate). Among the pre-proforma group, 0/30 patients had a fully recorded set of prehospital care observations; this improved to 9/15 (60%) in the proforma group.

## Conclusions

Improvements in trauma care are reliant on our ability to audit current practice, this in turn relies upon robust data collection. In a busy and stressful resuscitation room this is easily over looked. The introduction of standard documentation improves the clinician's ability to record such information.
